# Lung function, COPD and cognitive function: a multivariable and two sample Mendelian randomization study

**DOI:** 10.1186/s12890-021-01611-6

**Published:** 2021-07-22

**Authors:** Daniel H. Higbee, Raquel Granell, Gibran Hemani, George Davey Smith, James W. Dodd

**Affiliations:** 1 MRC Integrative Epidemiology Unit (IEU), University of Bristol, Oakfield Grove, Bristol, BS8 2BN UK; 2 Academic Respiratory Unit, Southmead Hospital, University of Bristol, Bristol, BS10 5NB UK

## Abstract

**Background:**

Observational studies show an association between reduced lung function and impaired cognition. Cognitive dysfunction influences important health outcomes and is a precursor to dementia, but treatments options are currently very limited. Attention has therefore focused on identifying modifiable risk factors to prevent cognitive decline and preserve cognition. Our objective was to determine if lung function or risk of COPD causes reduced cognitive function using Mendelian randomization (MR).

**Methods:**

Single nucleotide polymorphisms from genome wide association studies of lung function and COPD were used as exposures. We examined their effect on general cognitive function in a sample of 132,452 individuals. We then performed multivariable MR (MVMR), examining the effect of lung function before and after conditioning for covariates.

**Results:**

We found only weak evidence that reduced lung function (Beta − 0.002 (SE 0.02), *p*-value 0.86) or increased liability to COPD (− 0.008 (0.008), *p*-value 0.35) causes lower cognitive function. MVMR found both reduced FEV_1_ and FVC do cause lower cognitive function, but that after conditioning for height (− 0.03 (0.03), *p*-value 0.29 and − 0.01 (0.03) *p*-value 0.62, for FEV1 and FVC respectively) and educational attainment (− 0.03 (0.03) *p*-value 0.33 and − 0.01 (0.02), *p*-value 0.35) the evidence became weak.

**Conclusion:**

We did not find evidence that reduced lung function or COPD causes reduced cognitive function. Previous observational studies are probably affected by residual confounding. Research efforts should focus on shared risk factors for reduced lung function and cognition, rather than lung function alone as a modifiable risk factor.

**Supplementary Information:**

The online version contains supplementary material available at 10.1186/s12890-021-01611-6.

## Introduction

Cognitive function impacts important physical and mental health outcomes including mortality and educational attainment [1]. It exists on a continuum from normal cognitive function, to the potentially reversible state of mild cognitive impairment (MCI), which can lead to irreversible dementia [2, 3]. There are very limited therapeutic options which effectively increase cognitive function or treat MCI, so finding modifiable risk factors is important.

Lung function measures such as Forced Expiratory Volume in one second (FEV_1_) and Forced Expiratory Volume (FVC) have been linked with co-morbidities as early as the third decade [4]. There is also a growing body of evidence across cross sectional and longitudinal studies pointing to a an association between reduced lung function, Chronic Obstructive Pulmonary Disease (COPD) and cognitive function [5–10].

Co-morbid lung disease and cognitive impairment is associated with worse health outcomes, quality of life and increased healthcare utilisation [11]. The association between lung function and cognition may be due to shared risk factors seen more commonly in those with lung disease e.g. smoking [12, 13]. However associations independent of these factors mediated through plausible causal pathological pathways such as hypoxia, hypercapnia or chronic lung disease associated inflammation may also cause extra pulmonary end organ damage [5, 14].

Neuroimaging provides further evidence of a relationship between lung function and cognition. After adjustment for smoking, reduced lung function remains associated with white matter brain lesions [15], and a ‘dose response’ like relationship is seen between severity of lung function deficit and risk of cognitive impairment [6].

Mendelian randomization (MR) is a method which can overcome problems of unmeasured confounding and reverse causation typical of conventional observational epidemiology [16]. MR allows causal inference through the use of genetic variants as proxies for modifiable risk factors and health outcomes [17]. MR has multiple advantages, it is not influenced by behavioural or environmental factors and minimizes reverse causation [17]. Additionally, the effects are equivalent to lifetime differences, reducing issues relating to transient fluctuations in exposures [18]. Multivariable MR (MVMR) has further advantages, it includes multiple exposures in the model (e.g. lung function and educational attainment) allowing estimation of the direct causal effect of each exposure on the outcome. MVMR is a robust method when using two exposures that could act as a confounders [19]. MR has been used in this way to show that low FVC (but not FEV_1_) causes coronary artery disease [20].

Our objective was to use MR and MVMR to determine if lung function or liability to COPD causes lower general cognitive function. If the relationship is shown to be causal, then interventions to treat lung function could also be used to reduce subsequent cognitive decline or dementia.

## Methods

### Exposure populations

The largest available lung function Genome Wide Association Study (GWAS) by Shrine et al. was utilised, which reported 279 Single Nucleotide Polymorphisms (SNPs) to *p*-value < 5 × 10^−9^[21]. Lung function measurements used were FEV_1_, FVC, FEV_1_/FVC and Peak Expiratory Flow (PEF). The GWAS was adjusted for age, age^2^, height and smoking status [21].

For liability to COPD, we used 82 SNPs identified from a GWAS of 35,735 COPD and 222,076 control subjects [22]. Cases were defined as those with pre-bronchodilator spirometry of FEV_1_/FVC < 0.7 and FEV_1_ percent predicted < 80%. The GWAS was adjusted for age, age^2^, sex, height, principal components, and smoking. These two GWAS were used for 2-sample MR studies (2 sample MR is when the exposure and outcome GWAS are from two different sample populations).

The lung function GWAS methodology adjust for covariates of lung function and cognition e.g. height and smoking [12, 23]. This adjustment can lead to collider bias as SNPs can be related to lower levels of the covariates e.g. height, or to other adverse risk factors [18]. This can result in misleading SNP effect estimates and subsequent bias in MR studies [24]. To avoid these types of bias we performed MVMR using exposure SNPs discovered in GWAS that had not been adjusted for covariates. To find suitable exposure SNPs we performed a GWAS (adjusting for sex) based on 353,315 UK BioBank (UKBB) participants for “best measure” FEV_1_ and FVC. We did not adjust for age as this is not genetically determined and allele effects are independent of age. The SNPs discovered in the unadjusted GWAS were used in a two-sample MVMR model conditioning on SNPs for covariates: standing height, body mass index (BMI), current smoking. Educational attainment is a significant determinant of cognitive function, but it was not adjusted for in the outcome GWAS [25]. Therefore, we used SNPs for a GWAS of the UK Biobank variable “at what age did you complete your continuous full time education”, henceforth referred to as educational attainment. Participants were asked this via a touchscreen during assessment. By conditioning for educational attainment, we determined the direct effect of lung function on cognitive function. SNPs for these covariates were found in pre-existing GWAS performed in the UKBB [26]. See Additional files 1 and 2.

### Outcome population

Data from a meta-analysis of the Cohort of Heart and Aging Research in Genomic Epidemiology (CHARGE) and Cognitive Genomics Consortium (COGENT) was used [1]. UKBiobank participants were excluded from our analysis to ensure no overlap of exposure and outcome populations. This resulted in an outcome population of 132,452. The CHARGE and COGENT cohorts constructed a general cognitive function phenotype a number of cognitive tasks. The general cognitive function phenotype required the cohorts to have tested at least three different cognitive domains. As different tasks were used across the different sample populations, a consistent method of extracting general cognitive function was used. Principal component analysis was applied to the cognitive test scores to derive a measure of general cognitive function. The CHARGE and COGENT cohorts avoided taking more than one cognitive score from any individual cognitive test. GWAS Meta-analysis adjusting for age, sex and population stratification was performed using the derived measure of general cognitive function. Therefore, the outcome used in our analysis derived from the CHARGE and COGENT cohorts represents general cognitive function and is not domain specific. For full details of the tasks and samples see the supplementary information of the reference [1].

Both exposure and outcome populations were in those of European ancestry. For a summary of the UK Biobank population please see Additional file 1: Table e4.

### Statistical analysis

Statistical analysis was done using R Studio version 3.6.1 with MRCIEU/TwoSampleMR and MRInstruments packages [19, 27].

An assumption of Mendelian randomization is that the exposure SNPs are strongly associated with the exposure trait, in this case lung function and COPD. If the SNPs are only weakly associated with their respective exposure, this causes weak instrument bias and may invalidate the analysis. Therefore, F-statistics were calculated to assess exposure instruments strength (F statistic = beta^2^/standard error^2^). If the F-statistic is > 10 it means weak instrument bias in unlikely, and the higher the F-statistic the lower the chance of weak instrument bias [28]. For all exposures SNPs LD-clumping was performed using European reference population and the ieugwasr:ld_clump tool. This removes SNPs that are in close proximity (kb = 10,000), or highly correlated (r^2^ 0.001), to ensure that all SNPs used have independent effects. Steiger filtering was performed, to remove variants that caused more variance of the outcome than the exposure [27]. Duplicate SNPs were removed. Palindromic SNPs (i.e. A/T and C/G SNPs) with intermediate allele frequencies were excluded from analysis to ensure that the correct allele was being used. All SNPs were harmonised to ensure the exposure and outcome alleles were the same. See Additional files 1 and 2.

Inverse Variance Weighting (IVW) was used for main effect estimate for all analyses. IVW is a weighted regression of SNP-outcome on SNP-exposure associations combined.

MR assumptions and details of sensitivity tests used are detailed in the Additional files 1 and 2.

To account for the possibility of horizontal pleiotropy (where SNPs influence both exposure and outcome through independent pathways), in the 2S-MR analysis we performed MR Egger. Similar to IVW, MR-Egger is a weighted regression of SNP-outcome on SNP-exposure associations, but the intercept is not fixed to zero. We used weighted median and mode MR to minimise the effect of unbalanced instruments on an overall estimate of the mean. Heterogeneity (the variability in causal estimates obtained for each SNP) is an indication of potential violation of assumptions. This was calculated and assessed with a Q statistic, presented as a Q_*P*-value. MR Radial was performed to identify and exclude outliers and re-estimate IVW. MR radial removes SNPs that contribute more than 5% to the heterogeneity of the model [29].

IRB approval was not required as we did not recruit individuals but used publicly accessible data. UK Biobank is monitored by the UK Biobank Ethics and Governance Council.

## Results

The analysis using the adjusted lung function GWAS in a 2-sample lung function analysis is reported in Table 1 [21]. Effects are per standard deviation (SD) decrease in lung function measure. F statistic for all SNPs combined for each lung function trait were = 111, FEV_1_ = 69, FVC = 70, FEV_1_/FVC = 148, making weak instrument bias unlikely. The predictive causal effects show reduced FEV_1_ and FVC reduced cognition across all tests, but the evidence was weak (Table 1). All lung function measures combined did not show consistent direction of effect across the tests used. There was strong evidence of heterogeneity of effect based on the Q_*P*-value, especially when assessing all measures combined. However, there were no visual outliers (Additional file 1: Figures E1-E4) and minimal change in effect estimates after using MR-Radial to exclude outliers and recalculation of IVW. See Additional files 1 and 2.

**Table 1:** 2-Sample MR, decreasing lung function effect on cognitive function

Lung function measure	No. of SNPs	Test used	Cognitive function		
			Beta (SE)	*p*-value	Q_*P*-value
FEV_1_, FVC, FEV_1_/FVC, PEF	173	IVW	− 0.002 (0.02)	0.86	1.44 × 10^11^
		Weighted median	0.02 (0.02)	0.44	
		Weighted Mode	− 0.004 (0.03)	0.91	
		MR Egger	− 0.005 (0.03)	0.90	
FEV_1_	59	IVW	− 0.05 (0.04)	0.44	1.8 × 10^–8^
		Weighted median	− 0.02 (0.04)	0.46	
		Weighted mode	− 0.05 (0.08)	0.51	
		MR Egger	− 0.09 (0.1)	0.43	
FVC	68	IVW	− 0.004 (0.03)	0.87	0.01
		Weighted median	− 0.02 (0.04)	0.51	
		Weighted mode	− 0.03 (0.07)	0.65	
		MR Egger	− 0.16 (0.10)	0.13	
FEV_1_/FVC	93	IVW	0.01 (0.02)	0.52	0.01
		Weighted median	0.02 (0.02)	0.52	
		Weighted mode	0.007 (0.05)	0.87	
		MR Egger	0.02 (0.04)	0.67	

Negative beta indicates decreasing cognitive function

SE, standard error; Q_*P*-value, a measure of heterogeneity (*p*-value < 0.05 provides strong evidence of heterogeneity)

F statistic for COPD SNPs combined is 52, making weak instrument bias unlikely. There is a consistent direction of effect that increased liability to COPD causes lower cognition, however the strength of evidence is weak (Table 2). There was evidence of heterogeneity, however no outliers were identified visually (Additional file 1: Figures E5-E8) and exclusion of outliers in MR-Radial only minimally changed effect estimates.

**Table 2:** 2-Sample MR, COPD effect on cognitive function

Lung function measure	No. of SNPs	Test used	Cognitive function		
			Beta (SE)	*p*-value	Q_*P*-value
COPD	67	IVW	− 0.008 (0.008)	0.35	0.005
		Weighted median	− 0.01 (0.01)	0.16	
		Weighted mode	− 0.03 (0.02)	0.28	
		MR Egger	− 0.01 (0.02)	0.66	

Negative beta indicates decreasing cognitive function

### MVMR analysis

Using a threshold of *p* < 5 × 10^–8^, quality control and clumping the unadjusted GWAS of lung function in UKBB produced 360 SNPs for FEV_1_ and 464 SNPs for FVC explaining 3·6% and 4·8% of variance, respectively. Height (Beta 0.05 (SE 0.01), *p*-value < 0.001), BMI (− 0.1 (0.01), *p*-value < 0.001) and educational attainment (0.3 (0.07), *p*-value < 0.001) showed strong evidence for an effect on cognitive function. The SNPs discovered in the GWAS of current smoking only had very weak evidence of effect on cognitive function (− 0.04 (0.2), *p*-value 0.84), so they were not included in the analysis. The F-statistic for combined SNPs for each lung function trait are FEV_1_ = 38, FVC = 40, standing height = 50, BMI = 39 and age completed full time education = 1350, making weak instrument bias unlikely.

Results were calculated per SD decrease in lung function measure. There was strong evidence that reduced FEV_1_ (− 0.06 (0.03), *p*-value < 0.001) and FVC (− 0.06 (0.01), *p*-value < 0.001) cause lower general cognition, however with MVMR after conditioning with educational attainment the evidence became weak. Evidence also became weak after conditioning for height. This is probably due to the pleiotropy in the MR analysis as the unadjusted GWAS would have discovered SNPs that affected LF via height or educational attainment (Table 3).

**Table 3:** MVMR analysis results

Lung function measure	Condition	No SNPs (trait/condition)	Cognitive function	
			Beta (SE)	*p*-value
FEV_1_	None	298/0	− 0.06 (0.03)	< 0.001
FEV_1_	Height	212/299	− 0.03 (0.03)	0.29
FEV_1_	BMI	180/629	− 0.07 (0.03)	0.03
FEV_1_	Educational attainment	274/32	− 0.03 (0.03)	0.33
FVC	None	381/0	− 0.06 (0.01)	< 0.001
FVC	Height	278/314	− 0.01 (0.03)	0.62
FVC	BMI	224/595	− 0.05 (0.02)	0.004
FVC	Educational attainment	335/31	− 0.01 (0.02)	0.35

Effect of decreasing Lung function trait on cognitive function with and without conditioning for covariates

Negative beta indicates decreasing cognitive function

SE, standard error

## Discussion

Our analyses show weak evidence that lung function or liability to COPD causes lower general cognitive function. Most causal estimates show the same direction of effect as observational studies, with worse lung function, liability to COPD causing lower general cognitive function, however the evidence is weak. The observed association is likely secondary to residual confounding and collider bias. This would indicate that observed associations may be present but not causal [5–10].

Two-sample MR eliminates many confounders in observational epidemiology [17, 30]. We used a large number of robust lung function SNPs, and SNPs that influence liability to COPD, which have been well validated in large samples [21, 22]. MVMR was utilised to decrease the risk of bias and allowed us to condition for the effect of educational attainment. The effects of instrumental variables were assessed in a huge outcome population of similar age and the same ancestral population as our discovery GWAS.

Our analysis suggest that shared risk factors are likely explanations for the observed association between lung function and cognition, for example cigarette smoking. The short term effects of smoking on cognitive function are complex with acute nicotine consumption improving smokers’ cognition, and nicotine abstinence decreasing cognition [12]. Longitudinal research has shown that lower childhood IQ is associated with increased risk of smoking, and smokers have significantly worse cognition scores in old age than ex- or never-smokers [13]. Therefore, public health measures to reduce rates of smoking could improve both lung function and cognitive function.

Both cognition and lung function follow life course trajectories, influenced by a combination of genetic and life course factors [4]. Genetic determinants of lung development and disease are increasingly recognised [31]. In addition to shared environmental risk for lung function and cognitive function, shared genetic risks may be found. Genes involved in growth factors and Vitamin A regulation have been found to affect lung function. Both growth patterns and Vitamin A levels may have a role in cognitive function [23, 32]. It may be that genetic pleiotropy can determine both lung function and cognitive trajectories.

### Limitations

This study examines the effect of lung function and COPD on general cognitive function in general adult population. COPD has been shown to be associated with general cognitive function, but often more specific patterns of cognitive impairment including attention, memory, learning and motor function domains [5]. Therefore it may be that COPD has does have causal effect on these domains, not detected in our general cognitive function analysis.

This is comprehensive and robust analysis of causal association between lung function and risk of COPD, which uses two exposures and MVMR methodology. It is worth noting the SNPs in one of our two exposure analysis refers to ‘liability’ rather than confirmed diagnosis of COPD as no SNP guarantees future COPD, but MR is still a valid test of the causal null hypothesis for a binary exposure [33]. The mean age in our outcome sample was 56, lower than the average age of COPD diagnosis. The effects of SNPs for binary traits may be underestimated in the outcome sample, as participants have not yet developed COPD or its co-morbidities. However, the exposure SNPs were discovered and validated in large populations that had similar mean ages [21, 22]. Therefore the SNPs’ estimated effects on COPD liability should be accurate in the outcome population. If the mean age of the outcome population was older, it could introduce a survivor bias if a proportion of those with the SNPs had died from COPD and could not be recruited for the outcome GWAS [34].

One of the proposed mechanisms whereby lung function and COPD may cause reduced cognition is via cerebrovascular pathology [35]. Our outcome population excluded those with history of clinical stroke, this is unlikely to have excluded the proposed COPD specific brain changes, but would have excluded those with large vessel vascular damage causing changes in cognitive function.

Cognitive function can be correlated to factors that distort effect estimates. These include demography, (when a populations genetic variance is related to geographical location), assortative mating (partners are chosen due to phenotypes e.g. higher cognitive function, rather than randomly) and dynastic effects (phenotypic expression of parents genotype affects offspring phenotype e.g. parents with higher education giving educational books to their children) [36]. This can be corrected for by using within-family GWAS, a possible area for future studies [37]. However, MR studies have tended to over-estimate the effect of anthropomorphic traits which then attenuate when using within-family studies, and in this study we only found weak evidence of an anthropomorphic trait having an effect.

The populations used by the GWAS for exposure SNP’s only used European ancestry participants. These results may therefore not be generalizable to non-European ancestral populations. We did not have a replication cohort for the SNPs used in the MVMR analysis. The effects of the SNPs could have been over estimated due to “Winner’s Curse” phenomenon [38]. This occurs in GWAS when effect estimates are biased towards the SNPs with the strongest association. However, this phenomenon would bias results away from the null.

### Future research

Our outcome GWAS was performed with global cognitive function in the general population as a continuous outcome. Much research has focused on whether lung function and lung disease causes mild cognitive impairment (MCI) [39]. Normal cognitive function and MCI exist on a spectrum, but our study is unable to fully assess whether reduced lung function or lung disease causes MCI. If a GWAS of MCI becomes available MR could be used in future studies.

Our results indicate that lung function alone does not causes lower cognition in general population. However, lower lung function and lung disease have been shown to be associated with reduced cognitive function, but this is most likely due to shared risk factors. Research should focus on reducing exposure to these shared risk factors and optimising the management of co-morbidities in those with chronic lung disease.

## Conclusion

Our study shows that lung function and COPD do not cause reduced cognition in the general population. Previous observational studies suggesting a causal link were probably affected by residual confounding. The observed associations between reduced lung function, COPD and cognitive function remain important. Research should now focus on the management of cognitive impairment in these groups, rather than targeting lung function alone in order to improve cognition in the general population.

## Supplementary Information

**Additional file 1.** Supplementary information.

**Additional file 2.** SNP lists.

## Data Availability

All exposure SNPs used are available in the Additional file 2. They are also available online at the MRC IEU repository, https://gwas.mrcieu.ac.uk. ieu-b-106, ieu-b-105, ieu-b-104.
