# Supplementary material for: Lung function, COPD and cognitive function: a multivariable and two sample Mendelian randomization study
Source: BMC Pulm Med. 2021 Jul 22;21:246. doi: 10.1186/s12890-021-01611-6 (PMC8296721; doi:10.1186/s12890-021-01611-6)
Supplement: Supplementary file 1 — Additional file 1. Supplementary information. [file 12890_2021_1611_MOESM1_ESM.docx]

# Online Data Supplement

# Lung function, COPD and Cognition. A Multivariable & Two Sample Mendelian Randomization Study

Daniel H Higbee MBBS, Raquel Granell PhD, Gibran Hemani PhD, George Davey Smith FRS, James W Dodd MB ChB, PhD

## Contents

Appendix 1. Figures for 2 Sample MR, Lung function effect on cognition

Appendix 2. Figures for 2 Sample MR, COPD effect on cognition

Appendix 3. Results for 2 Sample MR, Lung function effect on cognition using MR Radial

Appendix 4. Results for 2 Sample MR COPD effect on cognition using MR Radial

Appendix 5. Explanation of assumptions, LD clumping, Steiger filtering and sensitivity analysis

Appendix 6. Summary of GWAS used

Appendix 7. Details for GWAS used for MVMR

## Appendix 1. Figures for 2 Sample MR, Lung function effect on cognition

### e-Figure 1. Scatter plot of SNP-effect on lung function trait and SNP-effect on Cognition

Each point on the graph represents the SNP-outcome association plotted against the SNP-exposure association. Bars indicate 95% confidence intervals. Coloured lines represent analysis method used. This shows no effect of lung function on cognition . MR Egger intercept is close to zero indicating no unbalanced directional pleiotropy.


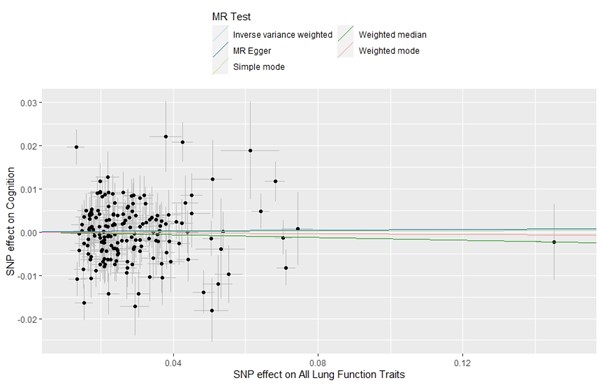


### e-Figure 2. Funnel plot of heterogeneity of causal effects of lung function traits on cognition

Each point is a SNP with its beta plotted against its inverse standard error. As the graph is funnel shaped, it indicates no heterogeneity.


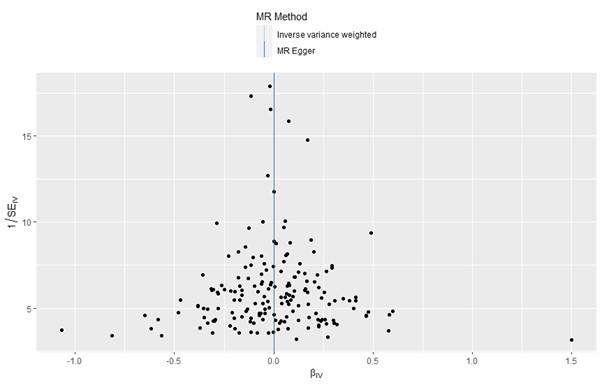


### e-Figure 3. Single SNP analysis of lung function traits on cognition

Each point represents individual SNP calculated effect size for lung function on the odds of cognition. Bars indicate 95% CI.


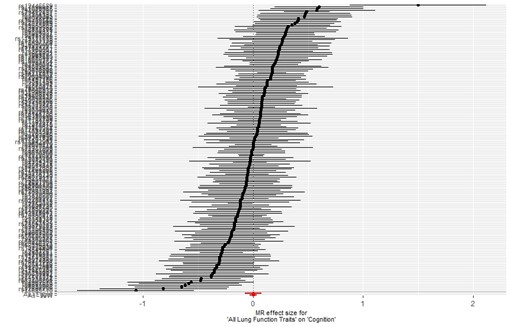


### e-Figure 4. Leave-one-out analysis of lung function traits on cognition

Each point represents the IVW estimate if the SNP on the y axis was left out of total analysis. Bars indicate 95% confidence intervals, demonstrating that no individual SNP is driving the causal effect estimate.


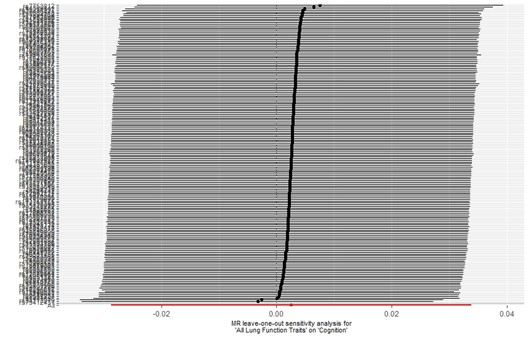


## Appendix 2. Figures for 2 Sample MR, COPD effect on cognition

### e-Figure 5. Scatter plot of SNP-effect of on COPD and SNP-effect on Cognition


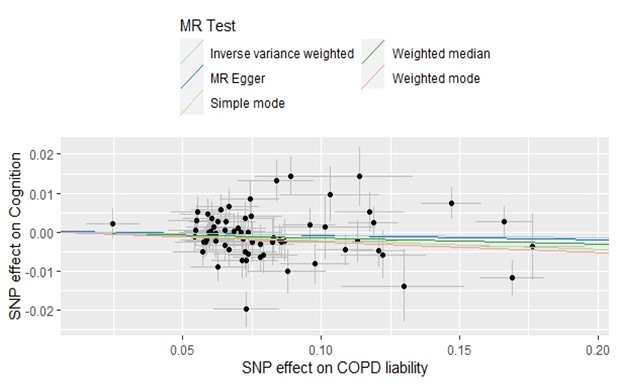


### e-Figure 6. Funnel plot of heterogeneity of causal effects of lung function traits on cognition


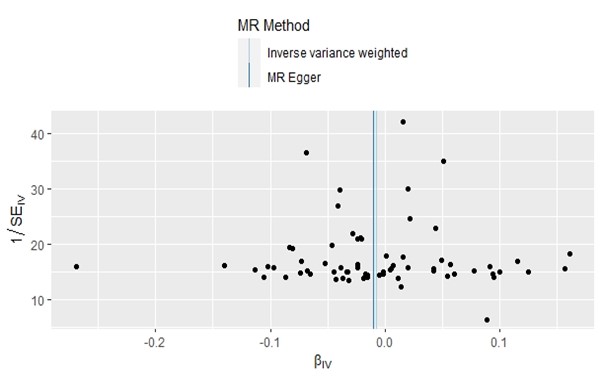


### e-Figure 7. Single SNP analysis COPD effect on cognition


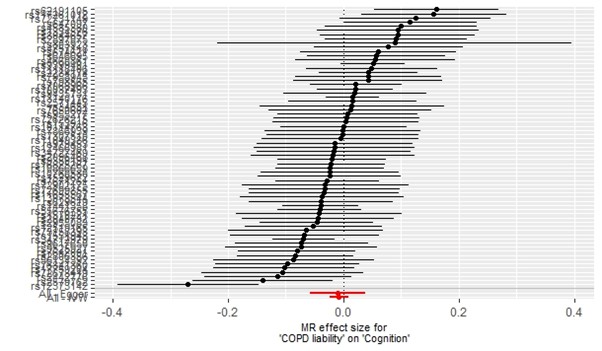


### e-Figure 8. Leave one out analysis COPD effect on cognition


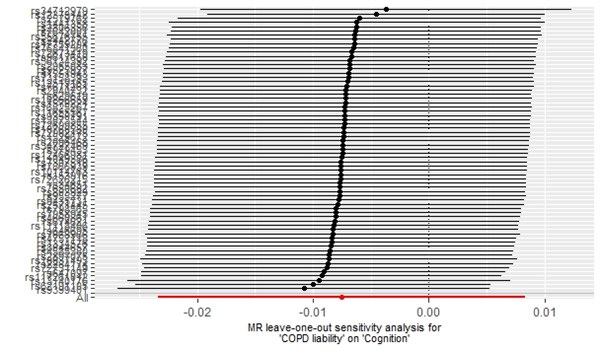


## Appendix 3. Results for 2 Sample MR all lung function SNPs (Shrine et al GWAS) effect on cognition using MR Radial

Using significance threshold for detecting 95% of outliers and modified second order weights 24 SNPs were excluded for being outliers. Results are after exclusion of these outliers

### e-Table 1. Results of 2 Sample MR all lung function SNPs (Shrine et al GWAS) effect on cognition using MR Radial after outlier exclusion

|  | All traits (Beta (SE)) | P-Value |
| --- | --- | --- |
| IVW (2^nd^ order weights) | -0.002 (0.01) | 0.90 |
| MR Egger | -0.02 (0.03) | 0.58 |

### e-Figure 9. MR Radial plots for 2 sample MR all lung function SNPs (Shrine et al GWAS) effect on cognition

Yellow dots represent outlier SNPs removed as explained over 5% heterogeneity. Blue dots represent SNPs kept in IVE Radial analysis. IVW casual estimate is close to zero and confidence levels cross zero indicating no strong evidence of an effect


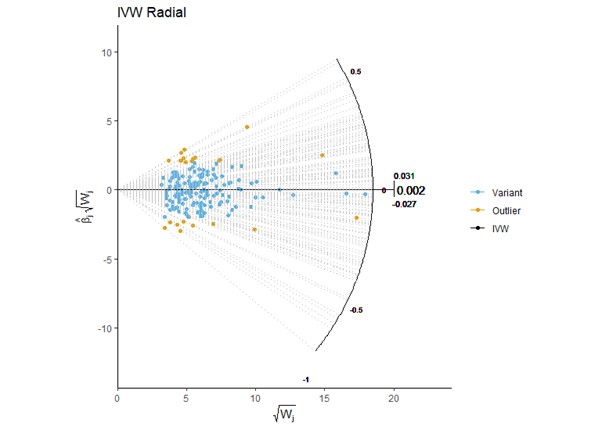


## Appendix 4. Results for 2 Sample MR COPD effect on cognition using MR Radial

Using significance threshold for detecting 95% of outliers and modified second order weights 8 SNPs were excluded for being outliers. Results are after exclusion of these outliers

### e-Table 2. Results of 2 Sample MR COPD effect on cognition using MR Radial after outlier exclusion

|  | All traits (Beta (SE)) | P-Value |
| --- | --- | --- |
| IVW (2^nd^ order weights) | -0.008 (0.007) | 0.30 |
| MR Egger | -0.001 (002) | 0.95 |

### e-Figure 10. MR Radial plot for 2 sample MR COPD effect on cognition


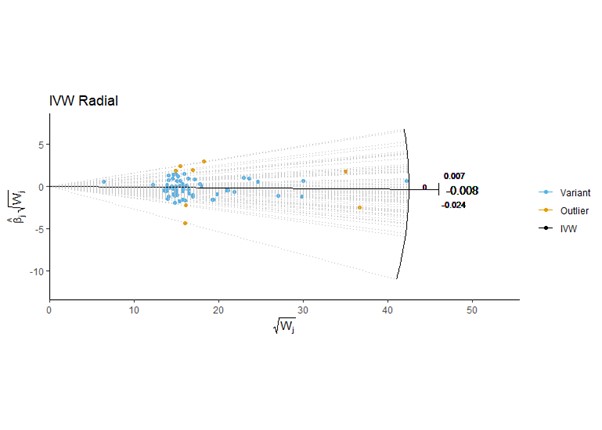


## Appendix 5. Explanation of assumptions and sensitivity tests, LD clumping, Steiger filtering and MR Radial

### Assumptions

We assume that our IVs have a true association with the exposures. This has been rigorously statistically tested in the discovery GWAS and effect estimation.[1, 2] F-statistic calculation shows that all exposure SNPs were unlikely to be weak instruments. In both GWAS used SNPs discovered are related to specific genes, cell types and biological pathways for lung tissue development.

We assume that our SNPs do not affect cognition except via their effect on LF/COPD, and that the SNPs have no associations with any confounders that are also associated with cognition. Although not possible to directly tests, our sensitivity and heterogeneity tests reduce the risk these assumptions were violated. To account for the possibility of horizontal pleiotropy (IVs affect multiple pathways) we performed MR Egger, weighted median and weighted mode tests. MR-Egger is similar to IVW except the y intercept is unconstrained. If the y intercept of the MR-Egger is not equal to zero then either there is unbalanced horizontal pleiotropy (the average pleiotropic effect differs from zero) or the pleiotropic effects are independent from the genetic association with the risk factor, or both.[3] Although power is lower compared to IVW, the gradient of the MR-Egger gives a causal estimate of the dose–response relationship between the genetic associations with the risk factor and those with the outcome, providing additional evidence for causal affect. To help avoid the effect of unbalanced instruments on an overall estimate of the mean by the IVW method, weighted median and mode MR methods were performed. A weighted median MR gives a consistent estimate of the causal effect when at least 50% of the weight comes from valid IVs, giving a greater robustness with strongly outlying causal estimates.[4] A weighted mode MR calculates an estimate based on the set of SNPs that form the largest homogenous cluster, which attempts to avoid the impact of invalid instruments.[5]

There was no evidence of population stratification (when subgroups within a sample are of different genetic ancestry) as assessed by linkage disequilibrium score regression in the original GWAS.

### LD-clumping

Linkage Disequilibrium (LD)-clumping, retains the most significant SNP at a locus to ensure all signals are independent. This was performed using the IEU stringent criteria of kb = 10000 and r^2^ = 0.001.

### Steiger Filtering

Steiger filtering estimates each SNP’s rsq.exposure and rsq.outcome in the outcome population.[6] Those SNPs that explain more variance in the outcome than exposure are excluded, as they could led to a reverse causal relationship. SNPs were removed if they explained more variance of the outcome than the exposure. SNPs were removed if they explained more variance of the outcome than the exposure.

### MR Radial

Heterogeneity in an IVW model may be caused by a small number of SNPs, or due to contribution from all SNPs. MR Radial sets a threshold that each SNP can contribute to overall heterogeneity (5% in our model) and uses iterative weights to reliable detect and removes these outliers.[7] The IVW can be re-estimated after exclusion of any detected outliers as a sensitivity test.

## Appendix 6. Comparison of GWAS used

### e-Table 3. Summary table comparing GWAS used

| Exposure | Data source | Sample size (% cases) | % European | GWAS Adjustment |
| --- | --- | --- | --- | --- |
| Lung function | UKBiobank & SpiroMeta | 400,102 | 100 | age, age^2^, sex, height, smoking status |
| COPD | 25 studies* | 257,811 (13.8%) | 100 | age, age^2^, sex, and height |
| FEV_1_  (MVMR) | UKBiobank | 345,590 | 100 | sex |
| FEV  (MVMR) | UKBiobank | 345,590 | 100 | sex |
| Height | UKBiobank | 461,950 | 100 | - |
| BMI | UKBiobank | 461,460 | 100 | - |
| Age completed fulltime education | UKBiobank | 307,897 | 100 | - |
| Outcome | Data source | Sample | % European | GWAS Adjustment |
| Cognition | COGENT, CHARGE | 132,452 | 100 | age, sex and population stratification |

*Please see supplementary table 1 of reference [2] for full table naming each study with description of respective: cases/controls number, smoking status, age, FEV_1_% and FEV_1_/FVC

The eight exposures in table e3 were drawn pre-dominantly, or entirely from UKBiobank. Demographics will vary slightly between each the exposures, due to missing data or different acceptability criteria. We present below a summary table of the UK Biobank population at the time they had lung function used for the “best measure” variables. This will closely correlate to the demographics for all exposure variables.

### e-Table 4. Summary of UKBiobank participants at time of lung function

| **Demographic** | **UK Biobank** |
| --- | --- |
| Age mean (SD) | 56.5 (8) |
| Sex (Female) | 54.4% |
| BMI, mean (SD) | 27.4 (5) |
| Never smoker | 53.6% |
| Ex-smoker | 36.2% |
| Current smoker | 10.2% |
| Pack year* Median (Interquartile range) | 19 (8 – 32) |
| Stroke | 1.3% |
| Diabetes | 4.5% |
| High blood pressure | 27.0% |

*for ex and current smokers only

## Appendix 7. Details of GWAS used for MVMR analysis

UKBiobank population and recruitment has been described in detail elsewhere.[8, 9] We used all individuals with valid genotype and results for the traits of interest. We did not look for or exclude those with specific underlying diseases. Interstitial lung disease is very unlikely to affect these results. Interstitial lung diseases are rare affecting <0.01% of UK adults (https://statistics.blf.org.uk/lung-disease-uk-big-picture). Within UKBiobank only 108 people are known to have idiopathic pulmonary fibrosis, of which 61 are receiving treatment. 1,768 people in UKBiobank report doctor diagnosed COPD, of which 1,277 are on treatment. As our sample size was >300,000 such small numbers will not skew the results.

Although not specifically tested, we do not believe there is any cross over between our exposure and outcome populations. All analysis was performed using BOLT LMM using the IEU GWAS pipeline. This uses a linear mixed model (LMM) to account for both relatedness and population stratification, therefore allowing a wider range of individuals to be included. A logistic regression using BOLT was performed for smoking and educational attainment. Only participants of European ancestry are used. A subset of 143,006 SNPs included in the model are directly genotyped. SNPs included are all meet the criteria:

Minor Allele Frequency >0.01

Genotyping rate >0.015

Hardy-Weinberg equilibrium p-value<0.0001

R^2^ threshold of 0.1

Pre-imputation is described in detail at below reference, rarer genetic variants were required to have high imputation scores; Info>0.3 for MAF >3%; Info>0.6 for MAF 1-3%; Info>0.8 for MAF 0.5-1%; Info>0.9 for MAF 0.1-0.5%

For full details please see references.[10-12]

### Forced Expiratory Volume in one second (FEV_1_)

GWAS performed on 345,590 participants.

Quantitative trait that was measured as litres to three decimal places.

Mean FEV_1_ = 2.853 (std = 0.780)

Estimated proportion of variance explained using inf model: 0.036

12,321,875 imputed SNPs in GWAS

44,522 SNPs reached significance at threshold of P_BOLT_LMM_INF <5x10^-8^

360 SNPs remained after LD-clumping

### Forced Vital Capacity (FVC)

GWAS performed on 345,590 participants

Quantitative trait that was measured as litres to three decimal places.

Mean FVC = 3.782 (std = 0.985)

Estimated proportion of variance explained using inf model: 0.048

12,321,875 SNPs imputed SNPs in GWAS

58,873 SNPs reached significance at threshold of P_BOLT_LMM_INF <5x10^-8^

464 SNPs remained after LD-clumping

All covariate GWAS were conducted by our colleagues at the IEU prior to this analysis being conducted. All are freely available on MRBase and at the IEU repository.[13, 14] Although not specifically tested, we do not believe there is any cross over between our exposure and outcome populations.

### Height

Standing height of 461950 UKBiobank participants was used. The GWAS was performed in 2018.[14]

Quantitative trait recorded as centimetres.

9851866 SNPs imputed in GWAS

241226 reached significance at threshold of P_BOLT_LMM_INF <5x10^-8^

990 SNPs remained after LD-clumping and removing a duplicate

### BMI

BMI of 461460 UKBiobank participants was used. The GWAS was performed in 2018.[14]

Quantitative trait recorded as Kg/m^2^

9851866 SNPs imputed in GWAS

68945 SNPs reached significance at threshold of P_BOLT_LMM_INF <5x10^-8^

799 SNPs remained after LD-clumping

### Current Smoking

Current smoking of 462434 UKBiobank participants was used. The GWAS was performed in 2018.[14]

Ordered categorical trait. We do not know exactly how many cases and controls this involved, but more recent UKBIOBANK figures show that there are 55666 current smokers, 197787 previous smokers, and 317645 never smokers. The GWAS we used is highly likely to reflect very similar proportions.

9851867 SNPs imputed in GWAS

1949 SNPs reached significance at threshold of P_BOLT_LMM_INF <5x10^-8^

37 SNPs remained after LD-clumping

Educational attainment

Age completed full time education of 307,897 UKBiobank participants was used. The GWAS was performed in 2018.

Ordered categorical trait.

9851867 SNPs imputed in GWAS

2026 SNPs reached significance at threshold of P_BOLT_LMM_INF <5x10^-8^

41 SNPs remained after LD-clumping

## Appendix 8. References

1. Shrine N, Guyatt AL, Erzurumluoglu AM, Jackson VE, Hobbs BD, Melbourne CA, Batini C, Fawcett KA, Song K, Sakornsakolpat P *et al*: **New genetic signals for lung function highlight pathways and chronic obstructive pulmonary disease associations across multiple ancestries**. *Nature Genetics* 2019, **51**(3):481-493.

2. Sakornsakolpat P, Prokopenko D, Lamontagne M, Reeve NF, Guyatt AL, Jackson VE, Shrine N, Qiao D, Bartz TM, Kim DK *et al*: **Genetic landscape of chronic obstructive pulmonary disease identifies heterogeneous cell-type and phenotype associations**. *Nature Genetics* 2019, **51**(3):494-505.

3. Bowden J, Davey Smith G, Burgess S: **Mendelian randomization with invalid instruments: effect estimation and bias detection through Egger regression**. *International journal of epidemiology* 2015, **44**(2):512-525.

4. Bowden J, Davey Smith G, Haycock PC, Burgess S: **Consistent Estimation in Mendelian Randomization with Some Invalid Instruments Using a Weighted Median Estimator**. 2016, **40**(4):304-314.

5. Hemani G, Bowden J, Davey Smith G: **Evaluating the potential role of pleiotropy in Mendelian randomization studies**. *Human Molecular Genetics* 2018, **27**(R2):R195-R208.

6. Hemani G, Tilling K, Davey Smith G: **Orienting the causal relationship between imprecisely measured traits using GWAS summary data**. *PLoS genetics* 2017, **13**(11):e1007081.

7. Bowden J, Del Greco M F, Minelli C, Zhao Q, Lawlor DA, Sheehan NA, Thompson J, Davey Smith G: **Improving the accuracy of two-sample summary-data Mendelian randomization: moving beyond the NOME assumption**. *International journal of epidemiology* 2018, **48**(3):728-742.

8. [**https://www.ukbiobank.ac.uk/**](https://www.ukbiobank.ac.uk/)

9. Trehearne A: **Genetics, lifestyle and environment**. *Bundesgesundheitsblatt - Gesundheitsforschung - Gesundheitsschutz* 2016, **59**(3):361-367.

10. Ruth Mitchell GH, Tom Dudding, Laura Corbin, Sean Harrison, Lavinia Paternoster: **UK BioBank Genetic Data: MRC-IEU Quality Control, version 2**. 2019.

11. Loh PR, Kichaev G, Gazal S, Schoech AP, Price AL: **Mixed-model association for biobank-scale datasets**. *Nat Genet* 2018, **50**(7):906-908.

12. Loh PR, Tucker G, Bulik-Sullivan BK, Vilhjálmsson BJ, Finucane HK, Salem RM, Chasman DI, Ridker PM, Neale BM, Berger B *et al*: **Efficient Bayesian mixed-model analysis increases association power in large cohorts**. *Nat Genet* 2015, **47**(3):284-290.

13. Hemani G, Zheng J, Elsworth B, Wade KH, Haberland V, Baird D, Laurin C, Burgess S, Bowden J, Langdon R *et al*: **The MR-Base platform supports systematic causal inference across the human phenome**. *eLife* 2018, **7**.

14. IEU: [**https://gwas.mrcieu.ac.uk/**](https://gwas.mrcieu.ac.uk/). 2020.
